# Exploring the role of extreme climate events (ECEs) in the incidence of respiratory disease in South Africa

**DOI:** 10.1007/s00484-025-03062-8

**Published:** 2026-01-12

**Authors:** Ogone Motlogeloa, Jennifer M. Fitchett, Neville Sweijd

**Affiliations:** 1 School of Geography, Archaeology and Environmental Studies, University of the Witwatersrand, Johannesburg, 2050 South Africa; 2 Alliance for Collaboration on Climate and Earth Systems Science (ACCESS), Council for Scientific and Industrial Research (CSIR), Pretoria, 0001 South Africa; 3 Department of Demography and Population Studies, University of the Witwatersrand, Johannesburg, 2050 South Africa; 4 BP012 Bernard Price Building, University of the Witwatersrand, 1 Jan Smuts Avenue, Johannesburg, Braamfontein 2000 South Africa

**Keywords:** Extreme climate events, Respiratory health, Medical claims, Seasonal patterns, Public health interventions, Seasonal preparedness

## Abstract

In recent decades, escalating extreme climate events (ECEs) have raised significant concerns regarding their effects on public health in South Africa, particularly respiratory illness. This study examined the relationship between ECEs and respiratory health outcomes over a 12-year period (2008–2019). A total of 48 ECEs were analyzed, of which 28 occurred in regions reporting more than 100 medical insurance claims for respiratory diseases. These events included storms, heatwaves, cold waves, floods, and tornadoes. Using a two-week lag period, we assessed their short-term association with respiratory claims. The findings revealed both increases and decreases in claims following ECEs, yet seasonal epidemiological trends exerted a more consistent and pronounced influence on respiratory health than individual extreme events. Percentage variations for statistically significant events ranged from approximately + 16% to + 121%, while decreases ranged from − 5% to − 178%. Although certain events displayed notable impacts, no distinct clustering was observed across seasons or years. These results underscore the importance of strengthening seasonal preparedness measures alongside climate-sensitive surveillance systems. Integrated approaches that address both seasonal and extreme climate risks are vital to safeguard vulnerable populations amid increasing climate variability in South Africa.

## Introduction

An extreme climate event (ECE) can be defined as a notable deviation from the historical mean or anticipated weather conditions within a specific geographic area, manifesting itself over a relatively limited temporal period, usually hours to days (Smith 2011; Van Der Walt and Fitchett 2022). These events exhibit distinctive features in terms of their intensity, duration, or frequency, and frequently have substantial ramifications for human societies, ecosystems, and the environment at large (Monirul 2003; Smith 2011). It is anticipated that climate change will increase the frequency and severity of ECEs globally, posing a significant danger to food security, water security, and economic stability in Southern Africa (Abrams et al. 2018; IPCC 2021; Lahsen and Ribot 2022). In Southern Africa, ECEs such as droughts, heat waves, and floods are becoming more frequent and severe (Davis-Reddy et al. 2017; Van Der Walt and Fitchett 2022). These events already have substantial effects on South Africa, including decreased agriculture yields, limited access to clean water, and property destruction (Apraku et al. 2021). The most vulnerable populations, including those living in rural communities and informal settlements, are often disproportionately affected (Curtis et al. 2017; Rother et al. 2020). ECEs have also been associated with an increase in the spread of infectious diseases and exacerbate existing health problems (Killingley, and Nguyen-Van‐Tam, 2013; McMichael 2015).

Infectious respiratory diseases are a major public health problem in South Africa due to the high prevalence of influenza and pneumonia (Tempia et al. 2020; Fraser et al. 2022), and the challenges posed by co-morbidities of HIV/AIDS and Tuberculosis (Bates et al. 2015). Influenza results in thousands of hospitalizations and fatalities annually (Duque et al. 2014; Tempia et al. 2019, 2020). Influenza typically has a seasonal pattern, with most cases occurring during the autumn and winter months in temperate regions of the world (Iuliano et al. 2018; Smit et al. 2020). In South Africa, the respiratory disease season spans 19 weeks (NICD 2019), spanning approximately May to August with considerably inter-annual and spatial variability (Motlogeloa et al. 2023). Common respiratory viral epidemics, such as influenza and respiratory syncytial virus (RSV), as well as concomitant bacterial pneumonia, display marked seasonal and interannual variability (Tellier 2006; Palache 2011; Weinberger 2021). The occurrence, total duration, and severity of infectious pandemics may become unpredictable as a result of climate change, including the effects of extreme climate events. (Towers et al. 2013). The intersection of climate hazards and pre-existing health vulnerabilities forms a critical axis of risk, whereby ECEs can magnify the health burdens of respiratory illnesses, particularly among those already immunocompromised or residing in low-resource settings.

ECEs can significantly impact the spread and incidence of diseases by disrupting environmental conditions and increasing exposure to pathogens (Matsumoto et al. 2012; McMichael 2015; Kontowicz et al. 2022). For example, floods can spread waterborne diseases, while overcrowding during emergency responses can facilitate respiratory transmission (Loevinsohn 1994; Beveridge 1991; Cavallo and Noy 2010). Heatwaves increase the risk of dehydration and respiratory stress, particularly in vulnerable populations (Campbell et al. 2018; Van Der Walt and Fitchett 2021a).

Although the direct mortality from ECEs is often recorded, their indirect impact on disease patterns—especially respiratory conditions—remains understudied in the African context (Fraser and Fitchett 2022). This study addresses that gap by analyzing medical aid claims for acute respiratory disease following ECEs in South Africa. By linking health outcomes to a wide variety of extreme climate events across multiple years and provinces, the study provides one of the first systematic examinations of climate-health linkages in the region using private health data as a proxy.

This study examines changes in respiratory disease burden following ECEs using proxy case data in the form of private-sector medical aid claims from Discovery Health Medical Scheme. While private medical insurance covers less than 20% of the South African population, primarily higher-income individuals, the dataset offers high-resolution, longitudinal information with consistent demographic fields (age and sex). Given the lack of publicly accessible national-level health data with comparable coverage and temporal depth, private claims serve as a pragmatic proxy for investigating post-ECE respiratory trends, while acknowledging inherent limitations in representativeness. Using the EM-DAT list of ECEs that have occurred in South Africa, coupled with Google News reports. The primary objective of this study is to assess whether there is a statistically significant change in the incidence of acute respiratory illness following documented extreme climate events. By bridging meteorological records with longitudinal health claims, this study not only addresses a critical data gap in South Africa but also contributes to the global understanding of how climate shocks affect respiratory disease dynamics, particularly in under-resourced or climatically vulnerable contexts.

## Materials and methods

### Study site

**Fig. 1:**
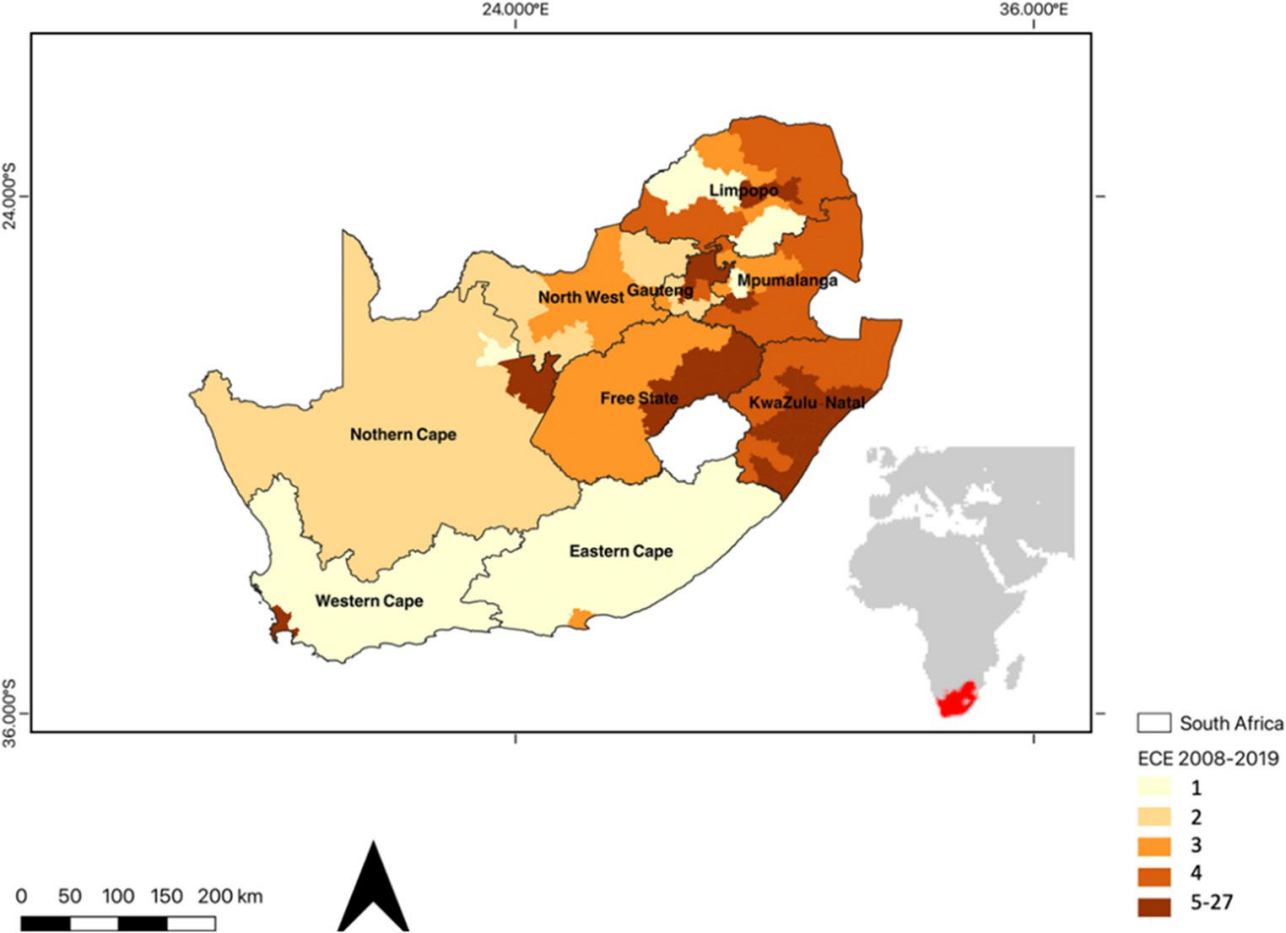
Map illustrating the number of ECEs that have occurred in South Africa between 2008–2019

South Africa is the southernmost country in Africa, situated between 22–35°S and 16–33°E. It has a land area of 1,220,000 km^2^ (Fig. 1) bordered by Botswana, Mozambique, Namibia, and Zimbabwe. A mountainous escarpment separates the coastal plain from a high plateau in the interior (Grab and Knight 2018). South Africa, with its diverse geography, experiences various extreme climate events (ECEs) across different regions. During the summer months, the interior areas, particularly the Northern Cape, Free State, and North West Province, are more susceptible to intense heatwaves, with temperatures often exceeding 30 °C and occasionally surpassing 40 °C (Fig. 1; Van Der Walt and Fitchett 2021a; LeComte 2020). In contrast, the southern coast and interior regions, including the Free State, Gauteng, and Mpumalanga province, are more prone to cold spells during the winter months, as cold fronts from the southern Atlantic Ocean bring colder air to these areas (Schulza and Maharaj 2007; Roffe et al. 2019; Lone and Ahmad 2020; Van Der Walt and Fitchett 2021b). Some South African locations also face significant flooding during the summer due to cut-off lows (COLs) and synoptic-scale baroclinic systems, while tornadoes are more common in the north-eastern regions, such as Mpumalanga, Gauteng, and KwaZulu-Natal, albeit relatively rare compared to other parts of the world (Fig. 1; Milford et al. 1994; Roffe et al. 2019; Grab and Nash 2023). Moreover, the country experiences recurring droughts, particularly in the western and central parts, characterized by semi-arid and arid conditions, resulting in significant impacts on agriculture, water resources, and the economy (Fig. 1; Zucchini and Adamson 1984; Rouault and Richard 2005; Sousa et al. 2018). The Day Zero drought in South Africa was one of the country’s most significant droughts, particularly impacting Cape Town (Wolski 2018; Ziervogel 2019).

Urban areas in South Africa, which are the focus of this study can experience particularly severe impact of ECEs. The presence of built infrastructure in cities and towns makes them vulnerable to destruction or damage during severe weather events, including floods, tornadoes, and storms. Additionally, the urban heat island effect can exacerbate heat waves, accelerating infrastructure deterioration and increasing energy demands for cooling (Salimi and Al-Ghamdi 2020). As urbanization continues to grow in South Africa, addressing the effects of ECEs on built environments becomes imperative for enhancing community resilience and promoting sustainable urban development.

## Data sources

### Case data

In this study, we analyzed two datasets to explore the correlation between extreme climate events (ECEs) and the incidence of respiratory diseases in South Africa. The primary dataset comprised a 12-year series (2008–2019) of medical insurance claims, specifically for individuals diagnosed with acute respiratory diseases as classified by ICD-10 codes J00 (Acute nasopharyngitis), J110 (Influenza with pneumonia), J111 (Influenza with respiratory infection), and J118 (Influenza with other infestations). This data was sourced from Discovery Health Medical Scheme and detailed daily claims from various municipalities, towns, and cities nationwide. Each record included the location of the claim, the filing date, and demographic details of the claimant such as age and gender. We aggregated the data weekly, starting each Sunday, and consciously excluded data post-2019 to prevent COVID-19 cases from distorting the analysis of seasonal and climatic trends (Motlogeloa et al. 2023). This approach also helps to reduce noise from daily fluctuations, weekends, and reporting lags. Weekly aggregation provides a clearer view of temporal trends and enhances comparability across events (NICD 2019). The research received ethical approval from the University of the Witwatersrand’s Human Health Research Ethics Committee (HREC), with the reference number M210617.

While it is recognized that medical aid coverage in South Africa extends to less than 20% of the population as of 2022. The dataset of medical insurance claims used in this study serves as a valuable proxy for monitoring trends in respiratory disease following extreme climate events (ECEs). However, it is acknowledged that trends observed among insured individuals may either underestimate or overestimate true incidence patterns in the broader South African population, particularly among uninsured or underserved communities. This limitation is explicitly noted to reinforce the scientific transparency of the study and the cautious interpretation of the findings. While we acknowledge that additional sociodemographic factors, such as income level, housing quality, and comorbidities, also influence vulnerability to respiratory disease, these variables were not available in the anonymized dataset provided. Only age and gender were consistently recorded across the 12 years, and therefore, these were used as proxy indicators of demographic variation. This dataset, derived from a leading health insurance provider, captures a substantial volume of systematic data over 12 years, offering a robust foundation for analyzing temporal patterns and associations with ECEs.

The insured population’s claims data provide a useful proxy for broader public health trends, as insured individuals are geographically dispersed across municipalities, towns, and cities. While coverage is uneven, the increasing demographic diversity among insured members introduces valuable heterogeneity into the sample. However, the spatial distribution of claims also reflects disparities in healthcare access and insurance penetration, with urban centres such as Johannesburg, Pretoria, and Durban being disproportionately represented. To address this imbalance and improve analytical robustness, we excluded regions with fewer than 100 claims following an ECE. This threshold helps to minimize statistical noise and ensures more reliable comparisons across more densely represented locations. Moreover, claims data represent the full spectrum of symptomatic responses to acute upper respiratory infections, from mild symptoms requiring over-the-counter medication at a pharmacy, through to severe cases leading to hospitalization up to the level of intensive care units. Although the results reflect the health outcomes of insured individuals, these individuals are part of the wider community ecosystem that includes both urban and rural settings. Therefore, the patterns observed in this segment can offer important insights into the potential health impacts of ECEs at a larger scale. Comparisons of these medical aid claims against hospitalization data for Baragwanath Hospital, the largest hospital in Africa, confirm consistency in the trends in the seasonality of the data observed. As detailed in Motlogeloa et al. (2023), this was achieved through a time-series alignment and visual trend analysis comparing weekly incidence curves across both datasets.

### Extreme climate event data

The complementary dataset utilized in our investigation documented the occurrence, geography, and classification of extreme climate events (ECEs). This information was extracted from the Emergency Events Database (EM-DAT), which catalogues natural disasters dating back to 1900 and is curated by the Centre for Research on the Epidemiology of Disasters (CRED) at the Université Catholique de Louvain’s School of Public Health in Brussels, Belgium. EM-DAT compiles extensive details on disasters, including their nature, location, dates, affected populations, casualties, injuries, and financial impact. Its reliability and comprehensiveness make EM-DAT an indispensable resource for scholars, policy formulators, and humanitarian groups. It supports the monitoring and analysis of disaster patterns, assisting in the formulation of measures for disaster risk mitigation and management of disaster response initiatives.

The classification of extreme climate events (ECEs) in this study was guided by the typologies and criteria employed by the Emergency Events Database (EM-DAT). This approach departs from percentile-based methodologies (Jong et al. 2023; Perkins and Alexander 2013), which rely on statistical thresholds derived from long-term meteorological records. Instead, EM-DAT prioritizes events based on their observed socio-economic impacts, offering a more contextually grounded and policy-relevant framework for identifying ECEs (Panwar and Sen 2020; Rosvold & Buhaug 2021). By leveraging this classification system, we were able to capture a comprehensive set of high-impact events with demonstrated disruption potential, aligning our study with real-world exposures rather than theoretical climatic anomalies. This facilitated a comprehensive and targeted search for relevant ECEs within the South African context, spanning from 2008 to 2019.

Further enriching our methodology, we expanded our data gathering through Google News to include additional reports on ECEs, using keywords like ‘flood’, ‘drought’, ‘heatwave’, and ‘cold wave’. This approach allowed us to build on the EM-DAT records and address potential under-reporting issues, noted in studies by Rataj et al. (2016) and Van Der Walt and Fitchett (2021a). To effectively collect and manage this information, a custom web scraping tool was developed. This tool was designed with advanced algorithms capable of accurately identifying and extracting relevant news content about ECEs, which were then saved in a structured CSV format for detailed analysis. To ensure the reliability of these records, only events corroborated by at least two independent and reputable sources, such as News24 and confirmed for temporal accuracy using South African Weather Service (SAWS) records, were included in the final dataset.

The collected data underwent a rigorous cleaning process, including a manual review to verify the relevance and severity of the events. Articles were excluded if they lacked temporal or geographic specificity, were unrelated to South African climate events, or represented duplicate coverage of the same incident. This review step ensured that only valid, high-confidence ECEs were retained for analysis. This meticulous approach to data collection has resulted in a robust and comprehensive dataset, crucial for analyzing the impacts of ECEs on respiratory health outcomes accurately and comprehensively.

**Fig. 2:**
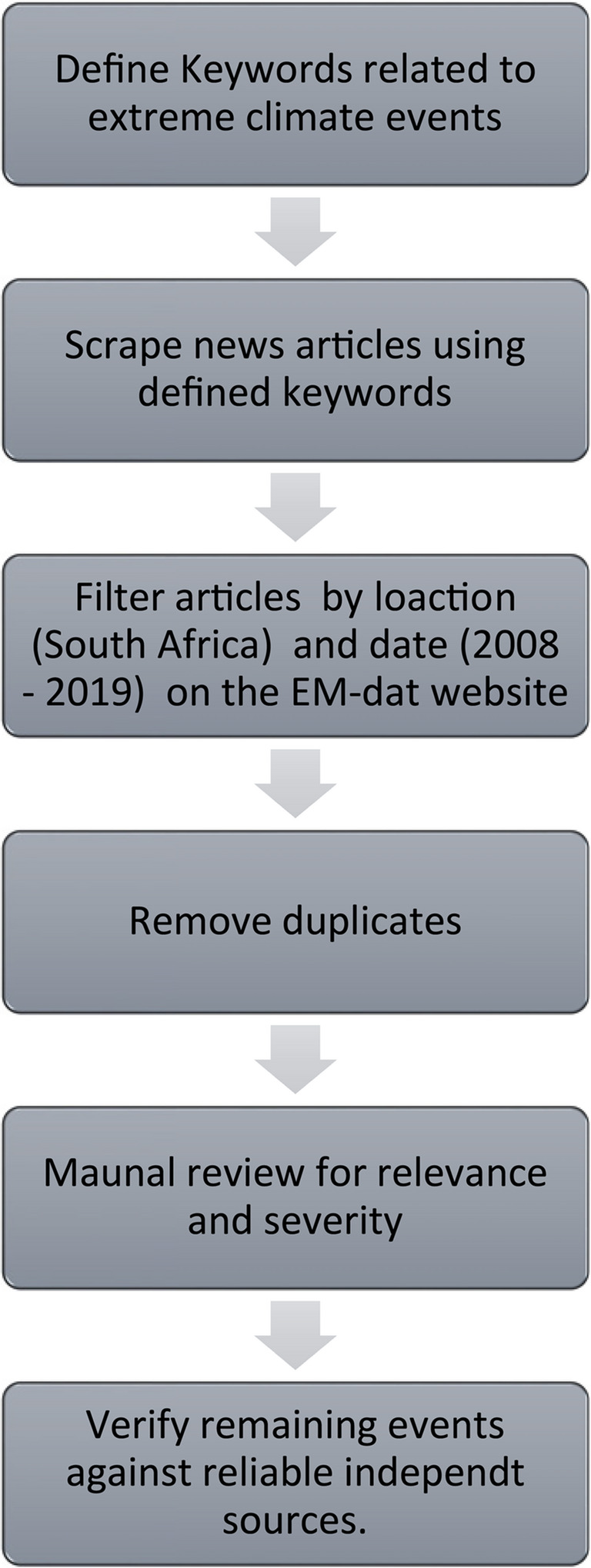
Flowchart illustrating the web scraping and filtering process for identifying relevant extreme climate events (ECEs)

To operationalise this component of the methodology, a custom web scraping tool was developed using Python to extract publicly available news articles from Google News spanning the period 2008 to 2019. The scraping protocol employed targeted keyword searches, such as “flood,” “drought,” “heatwave,” and “cold front”, in conjunction with Boolean logic to identify articles referencing weather-related disruptions within the South African context. Geographic specificity was achieved by filtering for provincial and municipal place names, while language filters restricted inclusion to English-language sources. Following initial extraction, a manual screening process was conducted to assess the temporal accuracy, geographic relevance, and thematic focus of each article. Events were only retained if corroborated by at least two independent and reputable sources (News24, TimesLIVE) and, where possible, cross-validated using bulletins or press releases issued by the South African Weather Service (SAWS). Duplicate entries were removed, and the final dataset was structured in comma-separated values (CSV) format, with fields capturing event type, date, location, severity notes, and verification sources. While the underlying scraping code cannot be made publicly available due to institutional restrictions, a methodological flowchart has been included in below (Fig. 2) to enhance transparency and facilitate potential replication:

### Data analysis

In our analysis of the claims data, we used a detrending process to isolate the impact of extreme climate events (ECEs) on respiratory diseases, independent of seasonal variations. We applied the Seasonal and Trend decomposition using Loess (STL) method within R, suitable for non-stationary time series data (Cleveland et al. 1990). Specifically, we used the stl() function in R with a periodicity of 52 (weekly data) and a seasonal window of 13 to account for quarterly variation while preserving short-term signals. This approach decomposed the series into trend, seasonal, and irregular components, with the trend component removed to stabilize the time series mean across the study period. The STL method was chosen for its non-parametric nature, allowing accurate modelling and removal of both linear and non-linear trends without assuming a fixed seasonal structure. The resulting detrended series was validated to confirm the absence of long-term patterns, enabling a focused analysis of ECE-related effects.

To quantify the impact of each ECE, we calculated the percentage deviation in respiratory disease claims from a baseline constructed using the same calendar weeks over the previous nine years (Roshan et al. 2022). A two-week post-ECE window was included to accommodate typical incubation periods and delayed onset of symptoms commonly observed in respiratory illnesses.

Inferential statistical analysis was then employed to assess the significance of observed deviations. Independent, two-tailed t-tests were applied to compare claim volumes during and after ECEs with the historical baseline, using a significance threshold of α = 0.05. We assessed assumptions of normality using the Shapiro-Wilk test. In cases where normality was violated, results were interpreted conservatively with attention to effect sizes.

**Fig. 3:**
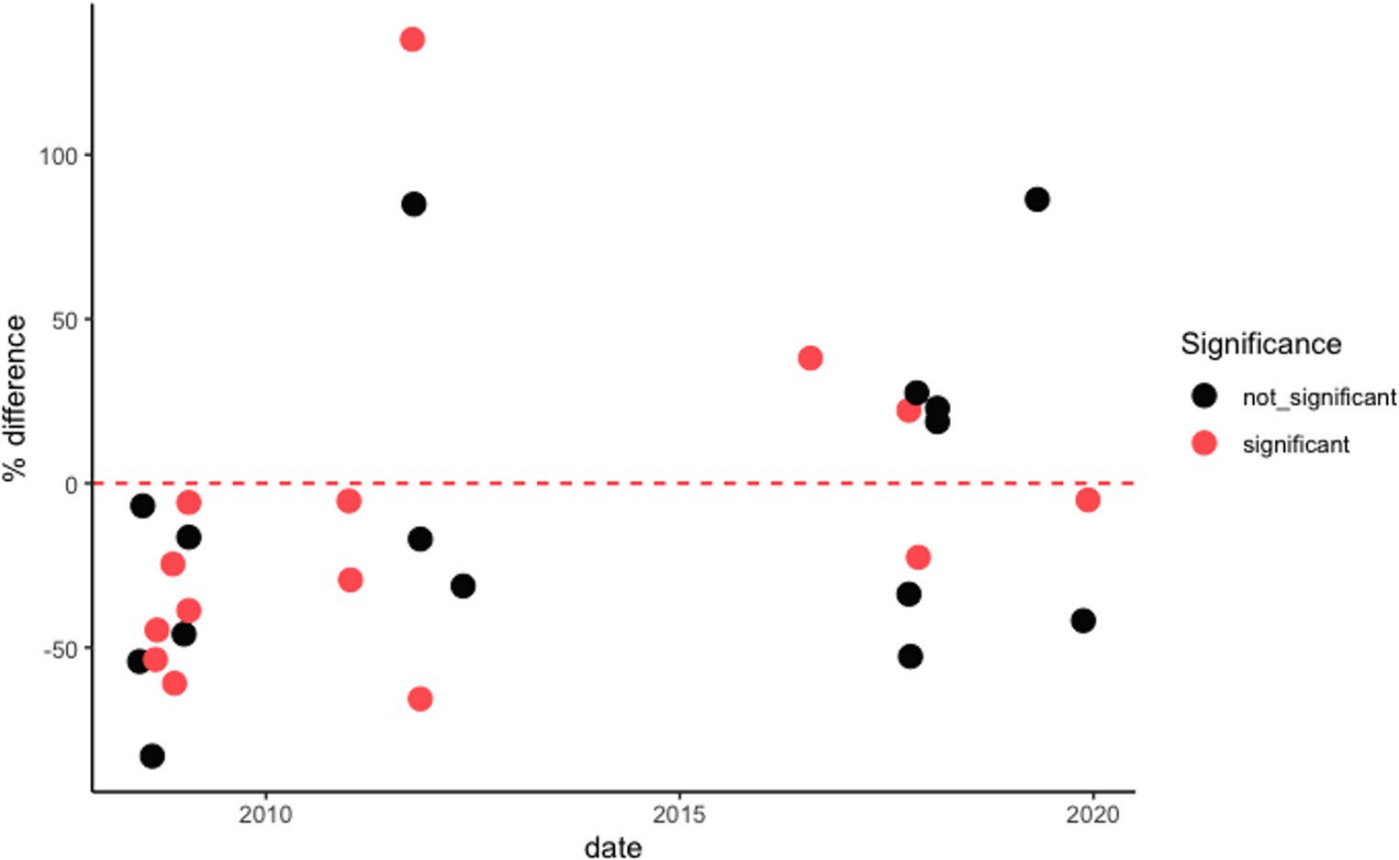
Significance of ECEs in South Africa from 2008–2019 at a two-week lag period

To mitigate the influence of statistical anomalies, we applied a ± 2 standard deviation threshold to the STL residuals, filtering out extreme outliers. The direction and magnitude of deviations both increases and decreases, are visualized in Fig. 3, with statistical significance indicated through colour-coded markers.

While multiple t-tests were conducted across numerous ECEs, no formal correction for multiple comparisons (e.g., Bonferroni adjustment) was applied due to the exploratory nature of the study and the heterogeneity of the events analyzed. Instead, interpretation was guided by the consistency of findings across event types, geographic regions, and time. Furthermore, we excluded weeks associated with major national holidays (e.g., Easter, December holidays) from baseline comparisons, due to known disruptions to health-seeking behaviour and clinical operations during these periods.

Finally, after establishing significance and filtering out unreliable locations (fewer than 100 claims per ECE), we conducted spatial and event-type specific comparisons to identify patterns in how different categories of ECEs impacted respiratory health outcomes.

## Results

### ECEs in South Africa 2008–2019

The EM-DAT database records 32 ECEs in South Africa between 2008 and 2019. Each of these events was covered in at least one news article captured by Google News. Excluding earthquakes, fires, and ecological extreme events, the dataset was reduced to 29 ECEs. An additional 19 ECEs were identified through Google News searches, including ten extreme temperature events and nine tornadoes. These were integrated with the 28 EM-DAT-based events to create a combined database of 47 ECEs (Table 1). This final set comprised droughts (3), tornadoes (9), heatwaves (5), cold waves (5), storms (11), and flooding events (14). The eThekwini district of KwaZulu-Natal experienced the highest number of storms and floods (4), while recurrent droughts were noted in the Western Cape, including the well-publicized ‘Day Zero Drought’ between 2008 and 2019. The mortality associated directly with these ECEs varied over the years; flood and storm events resulted in the greatest loss of life, resulting in a total of 363 deaths from 2008 to 2019 (Table 1). Among the 47 identified extreme climate events, 28 took place in regions where the cumulative number of Discovery Health Medical Scheme (DHMS) respiratory claims surpassed 100 during the 2008–2019 study period. Retaining only these events ensured adequate data density, improved statistical comparability, and minimised the influence of noise from under-represented areas.

**Table 1:** Overview of ECEs occurring in South Africa from 2008–2019. ***Note: “year_week” denotes the calendar year and corresponding epidemiological week (e.g.***,*** 2008_25 = week 25 of 2008)***

Year_ Week	Disaster Type	Location	Duration (days)	Source
2008_25	Cold wave	Cape Town (Western Cape); Gqeberha, East London (Eastern Cape); Durban (KwaZulu-Natal); Johannesburg (Gauteng)	7	Google News
2008_46	Storm	Durban City (KwaZulu-Natal)	1	EM-DAT, Google News
2008_47	Tornado	Molweni (KwaZulu-Natal)	1	Google News
2008_25	Flood	eThekwini district (KwaZulu-Natal)	1	EM-DAT, Google News
2009_1	Storm	Inchanga town - eThekwini district, Umzimkhulu area - Sisonke district, Richmond area - Umgungundlovu district (KwaZulu-Natal)	1	EM-DAT, Google News
2009_4	Heatwave	Johannesburg, Pretoria (Gauteng); Durban (KwaZulu-Natal); Cape Town (Western Cape)	1	Google News
2009_9	Flood	Kwadukuza (KwaZulu-Natal)	1	EM-DAT, Google News
2009_45	Tornado	Bulwer (KwaZulu-Natal)	1	Google News
2009_47	Storm	Cacadu district (Eastern Cape); Central Karoo district (Western Cape)	1	EM-DAT, Google News
2009_29	Flood	City of Cape Town district (Western Cape)	4	EM-DAT, Google News
2010_51	Storm	Free State, Mpumalanga, North West, Northern Cape, Limpopo, Gauteng, KwaZulu-Natal, Eastern Cape	2	EM-DAT, Google News
2011_1	Flood	Buffalo City, Amathole, Joe Gqabi, O. R, Tambo, Alfred Nzo, Chris Hani districts (Eastern Cape); Fezile Dabi, Thabo Mofutsanyane, Xhariep districts (Free State); City of Johannesburg, Ekurhuleni, Sedibeng, West Rand districts (Gauteng)	4	EM-DAT, Google News
2011_1	Flood	Amajuba, eThekwini, iLembe, Sisonke, Ugu, Uthukela districts (KwaZulu-Natal); Mopani, Sekhukhune, Vhembe, Waterberg districts (Limpopo); Frances Baard, Namakwa, Pixley ka Seme, Siyanda districts (Northern Cape); North West Province	4	EM-DAT, Google News
2011_41	Tornado	Duduza (Gauteng)	1	Google News
2011_41	Tornado	Fiksburg (Free State)	1	Google News
2011_54	Storm	KwaZulu-Natal	1	EM-DAT, Google News
2011_41	Storm	Meqheleng area-Ficksburg, Thabo Mofutsanyane district (Free State); Duduza town-Ekurhuleni district (Gauteng)	1	EM-DAT, Google News
2011_46	Heatwave	Johannesburg, Pretoria (Gauteng); Polokwane (Limpopo)	16	Google News
2011_47	Cold wave	Kimberley (Northern Cape); Cape Town (Western Cape); Gqeberha (Eastern Cape)	8	Google News
2012_1	Storm	Limpopo, Mpumalanga	1	EM-DAT, Google News
2012_19	Cold wave	Cape Town (Western Cape); Gqeberha (Eastern Cape); Kimberley (Northern Cape)	7	Google News
2012_25	Tornado	Bethlehem, Deneysville (Free State)	1	Google News
2012_43	Flood	Bathurst, Port Alfred, Kenton-on-Sea, Grahamstown (Eastern Cape)	5	EM-DAT, Google News
2013_22	Storm	Bishop Lavis, Hout Bay, Gugulethu, Strand, Khayelitsha, City of Cape Town (Western Cape)	1	EM-DAT, Google News
2014_9	Flood	Gauteng, KwaZulu-Natal, Limpopo, Mpumalanga, North West	1	EM-DAT, Google News
2014_46	Heatwave	Johannesburg, Pretoria (Gauteng); Polokwane (Limpopo); Kimberley (Northern Cape)	7	Google News
2015_1	Drought	KwaZulu-Natal, Free state, Limpopo, Mpumalanga, North-West, Cape Town (Western Cape)	~ 730	EM-DAT, Google News
2016_1	Heatwave	Ngaka Modiri Molema (North West)	9	EM-DAT, Google News
2016_31	Flood	Cape Town, Phillipi, Khayelitsha (Western Cape); Durban, Cato Manor, Amanzimtoti, Inanda, Ntuzuma, KwaMashu, uMlazi, Yellow Wood Park, Chatsworth -eThekwini Metropolitan Municipality, Ugu district (KwaZulu-Natal)	1	EM-DAT, Google News
2016_31	Tornado	Tembisa (Gauteng)	1	Google News
2016_46	Flood	Gauteng, KwaZulu Natal	1	EM-DAT, Google News
2016_51	Tornado	Standerton (Mpumalanga)	1	Google News
2016_52	Tornado	Standerton (Mpumalanga); Vaal Marina (Gauteng)	1	Google News
2017_23	Storm	Kraaifontein, Lavender Hill, Strand, Kalkfontein, Delft, Mfuleni, Mandalay, Hout Bay, Kraaifontein, Cape Town, Rheenendal, Welbedacht, Knysna, Pacaltsdorp, Mossel Bay, George to Plettenberg Bay -Eden, Welbedacht - Cape Winelands (Western Cape)	1	EM-DAT, Google News
2017_41	Storm	Durban, Bluff, Jacobs, Montclair, Glenwood, Umlazi, Merebank, Isipingo, Nquthu, Umzinyathi (KwaZulu-Natal); Johannesburg, Ekurhuleni-West Rand District Municipality (Gauteng)	1	EM-DAT, Google News
2017_18	Drought	Cape Town, Cape occidental, Cape oriental, North Cape (Western Cape)	~ 365	EM-DAT, Google News
2017_46	Cold wave	Johannesburg, Pretoria (Gauteng)	7	Google News
2017_20	Flood	Kquthu-Umzinyathi District, Zinkwazi-Ilembe District, Mzingwenya-Uthungulu District, uMzumbe municipality, uMdoni, uMuziwabantu (KwaZulu-Natal)	5	EM-DAT, Google News
2018_6	Heatwave	Johannesburg, Pretoria (Gauteng); Klerksdorp, Potchefstroom (North West)	7	Google News
2018_25	Cold wave	Cape Town (Western Cape)	1	Google News
2019_10	Flood	KwaZulu-Natal, Eastern Cape, Free State	4	EM-DAT, Google News
2019_17	Flood	Ntuzuma, Verusalam, Inanda, KwaMashu (KwaZulu-Natal)	4	EM-DAT, Google News
2019_18	Flood	Eastern Cape	1	EM-DAT, Google News
2019_46	Storm	Inanda (KwaZulu-Natal)	1	EM-DAT, Google News
2019_1	Drought	Western Cape, Eastern Cape, Northern Cape	12	EM-DAT, Google News
2019_49	Flood	Pretoria (Gauteng)	9	EM-DAT, Google News
2019_51	Tornado	New Hanover (KwaZulu-Natal)	1	Google News

### ECEs and the incidence of acute respiratory diseases

Of the 47 ECEs (Table 1), only 28 occurred in regions which had more than 100 total DHMS medical aid claims and were thus retained for further analysis. Importantly, some ECEs affected more than one area, resulting in duplicate entries of the event where the local medical aid claim response is tested (Table 2). Extreme climate events, such as storms, heat waves, cold waves, floods, and tornadoes, have significant implications for public health and medical health insurance claims. To examine the association between these extreme climate events and medical health insurance claims, a comprehensive analysis was undertaken, incorporating a two-week lag period for each event. The findings from this study are summarized below.

**Table 2:** Changes in medical insurance claims relative to baseline in the event of ECEs

Municipality/City	Week	Year	Disaster Type	Province	Baseline Claims	Change in Claims	% Change from Baseline	95% CI for Change	*P* Value	Sd Claims Other Years	Sd Claims Year Test
Pretoria	49	2019	Flood	Gauteng	54	−3	−5	54	0,0004	1,87	0,65
Inanda	46	2019	Storm	KwaZulu-Natal	34	−14	−42	17	0,0549	1,55	0,78
Inanda	17	2019	Flood	KwaZulu-Natal	87	75	86	42	0,0773	3,98	3,12
Durban	41	2017	Storm	KwaZulu-Natal	113	−38	−34	42	0,0799	1,68	1,48
Johannesburg	41	2017	Storm	Gauteng	82	18	22	151	0,0007	1,79	0,72
Inanda	42	2017	Storm	KwaZulu-Natal	51	−27	−53	10	0,8318	1,71	1,96
Kempton Park	42	2011	Storm	Gauteng	21	17	85	12	0,1348	1,28	2,52
Johannesburg	1	2011	Flood	Gauteng	85	−5	−5	31	< 0.0001	1,48	0,79
Durban	2	2011	Flood	KwaZulu-Natal	135	−40	−29	58	0,0180	2,45	1,51
Durban	46	2008	Storm	KwaZulu-Natal	113	−28	−25	54	0,0109	1,87	1,28
Durban	25	2008	Flood	KwaZulu-Natal	328	−178	−54	37	0,3030	4,36	2,42
Pinetown	27	2008	Flood	KwaZulu-Natal	23	−2	−7	10	0,7008	2,17	1,51
Durban	1	2009	Flood	KwaZulu-Natal	139	−64	−46	26	0,9264	2,29	1,29
Pretoria	4	2009	Heatwave	Gauteng	98	−38	−39	58	< 0.0001	2,51	1,36
Johannesburg	4	2009	Heatwave	Gauteng	117	−7	−6	67	0,0412	1,88	2,02
Durban	4	2009	Heatwave	KwaZulu-Natal	150	−25	−16	59	0,6856	2,36	1,70
Johannesburg	46	2011	Heatwave	Gauteng	72	−12	−17	43	0,2096	1,21	0,89
Pretoria	46	2011	Heatwave	Gauteng	61	−40	−66	16	0,0144	2,01	0,87
Johannesburg	6	2018	Heatwave	Gauteng	143	27	19	98	0,0515	2,27	1,81
Pretoria	6	2018	Heatwave	Gauteng	103	23	23	62	0,8234	2,49	1,55
Gqeberha	33	2008	Coldwave	Eastern Cape	29	−24	−83	2	0,1831	2,15	1,15
Durban	35	2008	Coldwave	KwaZulu-Natal	237	−127	−54	43	0,0159	2,93	1,67
Johannesburg	36	2008	Coldwave	Gauteng	226	−101	−45	137	< 0.0001	3,23	1,22
Gqeberha	21	2012	Coldwave	Eastern Cape	42	−13	−31	15	0,1089	3,74	2,13
Pretoria	46	2017	Coldwave	Gauteng	56	16	28	35	0,5330	1,83	2,90
Johannesburg	47	2017	Coldwave	Gauteng	65	−15	−23	83	< 0.0001	1,20	0,50
Johannesburg	31	2016	Tornado	Gauteng	319	121	38	58	0,0002	3,94	5,93
Kempton Park	41	2011	Tornado	Gauteng	24	32	135	12	0,0081	1,40	2,67
Durban	47	2008	Tornado	KwaZulu-Natal	115	−70	−61	149	< 0.0001	1,94	0,46

A subset of storm events correlated with significant fluctuations in medical health insurance claims, as reflected by p-values ranging from < 0.001 to 0.1, detailed in Table 2. These events resulted in a range of insurance claim adjustments, with increases between 16% and 121% and decreases from − 5% to −178%. The incidences of these storm events were distributed across various locations and spanned from 2008 to 2019, exhibiting no discernible seasonal or temporal trends. Notably, the standard deviations for claims in other years and the standard deviations for claims in the test year provide additional context, indicating the variability of claims associated with each event, as seen in the entries for each city and event in Table 2. For instance, the storm in Inanda in 2019 showed a standard deviation of 1.55 for other years and 0.78 for the test year, highlighting the variability in claim amounts.

Similarly, heatwave events were associated with notable variations in insurance claims, evidenced by p-values in the same statistical range. Heatwaves led to a rise in claims by 18% to 27% and a reduction by −7% to −40%, without a focused spatial or temporal distribution. The standard deviations in claims for other years and in the test year for these events show the fluctuations in claim amounts during heatwaves, providing insight into the variability of claims, which ranges from 0.72 to 2.51 for other years and 0.89 to 2.02 for the test year. Cold wave events were linked to significant changes in claims, with increases noted from 16% to 85%, and decreases ranging from − 83% to −178%. The occurrence of significant cold wave events did not show any regular patterns in their distribution. The standard deviations for claims during cold wave events demonstrate considerable variability, with figures ranging from 0.50 to 3.90 for other years and 1.15 to 2.90 for the test year, underscoring the unpredictable nature of insurance claims related to cold waves.

Flood-related events also showed a significant correlation with changes in insurance claims, with p-values ranging from < 0.001 to 0.1. These events accounted for claim increases from 75% to 86% and decreases ranging from − 5% to −178%, occurring without a consistent pattern in location or timing. The standard deviations for these events highlight the variability in claims, with figures ranging from 0.65 to 3.12 for claims in the test year and 1.48 to 4.36 for claims in other years, emphasizing the unpredictable nature of insurance claims associated with flood events. Tornado events displayed a similar trend, with significant impacts on insurance claims ranging from positive changes of 38% to 135% to negative changes between − 61% to −70%. Like other ECEs, tornado events did not present any particular spatial or temporal patterns. The variability in claims related to tornadoes is evident in the standard deviations, with figures ranging from 0.46 to 5.93 for claims in the test year and 1.40 to 3.94 for claims in other years. These statistics underscore the substantial fluctuations in insurance claims associated with tornado occurrences.

The results, detailed in Fig. 3; Table 2, consistently reveal no statistically significant relationships between medical health insurance claims and extreme climate events. While some events showed statistically significant associations, both positive and negative, there were no discernible trends or patterns regarding spatial or temporal clustering. For example, the flood in Pretoria in 2019 and the tornado in Durban in 2008 both show p-values well below 0.05, indicating statistically significant impacts on insurance claims for those specific weeks.

However, when considering broader trends within groups of similar ECEs (like all floods or all heatwaves across multiple events and years), the data does not show consistent trends that reach statistical significance. This suggests that while individual events can have significant impacts, these effects do not uniformly manifest across all occurrences of the same type of ECE. These findings emphasize the complexity of the relationship between extreme climate events and medical health insurance claims, warranting further research and consideration for policy and healthcare planning (Fig. 2). The comprehensive analysis presented provides valuable insights into the diverse impact of extreme weather events on the health insurance system.

## Discussion

### Variation in medical claim patterns following extreme climate events (ECEs)

The principal observation of this study is that, overall, there was no consistent pattern of statistically significant changes in medical health insurance claims for respiratory disease following extreme climate events (ECEs). While some events were associated with measurable changes in claim volumes, these effects were heterogeneous, manifesting as both increases and decreases in different contexts, and not consistent across all ECE types, locations, or seasons (Table 2; Fig. 2). This absence of uniformity suggests a complex interplay of contextual and environmental factors that influence the relationship between ECEs and respiratory health outcomes.

To understand these varied results, it is essential to explore how ECEs may affect healthcare-seeking behaviours and access. Extreme conditions such as flooding, cold snaps, or heatwaves may hinder individuals’ ability or willingness to visit healthcare providers, which in turn could suppress claim volumes even if disease incidence increases. Conversely, some ECEs may create conditions that facilitate viral transmission through overcrowding, exposure to allergens or pollutants, or decreased immune function, but the effects may differ widely depending on timing, geographic region, and population vulnerability.

ECEs may also create temporary alterations in local meteorological conditions that influence viral survival and transmission (Iwasaki and Pillai 2014). For instance, storms and floods may increase exposure to respiratory irritants, while cold weather events can exacerbate underlying respiratory conditions by irritating airways (Carmona et al. 2016). Human behavioural responses, such as crowding in shelters or staying indoors with limited ventilation, can also affect exposure patterns (Gaillat et al. 2008; Kontowicz et al. 2022). Furthermore, stress associated with extreme events may temporarily weaken immune responses, compounding susceptibility to respiratory infections.

A crucial limitation of this study is the representativeness of the dataset, which includes only private-sector medical insurance claims, covering less than 20% of the South African population, predominantly higher-income individuals. Vulnerable populations, including those in lower-income brackets and informal settlements, are more likely to experience adverse health outcomes following ECEs, yet are underrepresented in this dataset. Their limited access to private healthcare and insurance means that their respiratory health burdens may be overlooked in such analyses. This limitation may partially explain the absence of consistent patterns in our findings and highlights the importance of integrating public health system data in future research. Addressing this limitation will not only enhance the equity of health surveillance but also help tailor climate-health responses to at-risk communities.

### Implications for respiratory disease preparedness

The findings of this study highlight the variability in the impact of extreme climate events (ECEs) on respiratory disease claims, reinforcing the assertion that seasonal patterns of respiratory illness exert a more significant influence than individual ECEs (Motlogeloa et al. 2023). Consequently, healthcare preparedness initiatives should prioritise managing the direct effects of ECEs, such as heatstroke during heatwaves, hypothermia during cold snaps, and trauma associated with tropical cyclones (Campbell et al. 2018; Codjoe and Atiglo 2020). While ECEs may influence respiratory health outcomes, these effects are often overshadowed by dominant seasonal epidemiological trends (Forshee-Hakala 2015; Kimambo 2018).

However, in the context of ongoing climate change, which is expected to amplify both the frequency and severity of ECEs, continuous monitoring of their impact on respiratory health remains imperative (Onuma et al. 2017). According to the Intergovernmental Panel on Climate Change (IPCC), the Southern African region is projected to experience increased temperature extremes, prolonged droughts, and more intense storm systems over the coming decades. These projections suggest heightened vulnerability to climate-sensitive health outcomes, including respiratory illnesses. The intensification of ECEs may also produce novel interactions between meteorological conditions and respiratory pathogens, for example, increased air pollution during droughts may exacerbate respiratory inflammation, while overcrowding in shelters during flood-related evacuations could facilitate viral transmission (Guha-Sapir et al. 2016; Codjoe and Atiglo 2020).

Furthermore, the variability in medical claim responses to ECEs underscores the importance of contextual factors such as human behaviour, pre-existing health status, and environmental exposures. These interrelated elements necessitate sophisticated surveillance systems and preparedness strategies tailored to specific population needs. It is particularly important to acknowledge the limitations of the dataset used in this study, which draws exclusively from private-sector health insurance claims. This dataset captures less than 20% of the South African population, predominantly higher-income individuals, and omits a substantial segment of the population that may be more vulnerable to ECE-related health outcomes (Motlogeloa et al. 2023). Populations without access to private medical coverage may face distinct barriers to care during ECEs and may experience disproportionate respiratory burdens.

Given this limitation, future research should aim to integrate public-sector health data and strengthen targeted surveillance in under-resourced communities. Such an approach would enhance the equity and representativeness of health impact assessments and enable the development of more inclusive and effective climate-health preparedness strategies. Integrating data from both public and private healthcare systems will provide a more comprehensive understanding of ECE-related respiratory risks and support evidence-based policy interventions that prioritise at-risk communities.

## Conclusion

This study investigated the association between extreme climate events (ECEs) and respiratory disease claims in South Africa using private-sector medical insurance data. The primary objective was to determine whether statistically significant changes in respiratory claims occurred following ECEs and to assess the consistency of these effects across time, regions, and event types.

Although certain ECEs were linked to fluctuations in respiratory claims, no uniform spatial or temporal pattern was observed. These findings suggest that the health impacts of ECEs are heterogeneous and context-dependent, influenced by interacting factors such as environmental exposure, population vulnerability, and healthcare access. In contrast, broader seasonal trends exerted a stronger and more consistent influence on respiratory health, underscoring the importance of strengthening seasonal preparedness within public health strategies.

Future research should expand climate-sensitive health surveillance by integrating private- and public-sector health data to improve representativeness and inform evidence-based planning. Enhanced early warning systems, seasonal vaccination campaigns, and targeted support for vulnerable populations can help reduce the respiratory health burden associated with both seasonal and extreme climate conditions.

By highlighting the interplay between climatic variability and health outcomes, this study contributes to the evidence base required to design more resilient, responsive, and equitable health systems in South Africa.

## Data Availability

Data supporting the findings of this study are not available.

## Code Availability

The code used in this study is not available.
